# Systematic identification and characterization of *Aedes aegypti* long noncoding RNAs (lncRNAs)

**DOI:** 10.1038/s41598-019-47506-9

**Published:** 2019-08-21

**Authors:** Azali Azlan, Sattam M. Obeidat, Muhammad Amir Yunus, Ghows Azzam

**Affiliations:** 1 School of Biological Sciences, Universiti Sains Malaysia, 11800 Penang, Malaysia; 2 Infectomics Cluster, Advanced Medical & Dental Institute, Universiti Sains Malaysia, Bertam, 13200 Kepala Batas, Penang Malaysia

**Keywords:** Long non-coding RNAs, Transcriptomics, Gene regulatory networks

## Abstract

Long noncoding RNAs (lncRNAs) play diverse roles in biological processes. *Aedes aegypti* (*Ae*. *aegypti*), a blood-sucking mosquito, is the principal vector responsible for replication and transmission of arboviruses including dengue, Zika, and Chikungunya virus. Systematic identification and developmental characterisation of *Ae*. *aegypti* lncRNAs are still limited. We performed genome-wide identification of lncRNAs, followed by developmental profiling of lncRNA in *Ae*. *aegypti*. We identified a total of 4,689 novel lncRNA transcripts, of which 2,064, 2,076, and 549 were intergenic, intronic, and antisense respectively. *Ae*. *aegypti* lncRNAs share many characteristics with other species including low expression, low GC content, short in length, and low conservation. Besides, the expression of *Ae*. *aegypti* lncRNAs tend to be correlated with neighbouring and antisense protein-coding genes. A subset of lncRNAs shows evidence of maternal inheritance; hence, suggesting potential role of lncRNAs in early-stage embryos. Additionally, lncRNAs show higher tendency to be expressed in developmental and temporal specific manner. The results from this study provide foundation for future investigation on the function of *Ae*. *aegypti* lncRNAs.

## Introduction

Long noncoding RNAs (lncRNAs) are arbitrarily characterised as RNA molecules greater than 200 nucleotides in size that do not encode proteins^1,2^. Although lncRNAs lack coding potential, they undergo post-transcriptional modifications similar to coding mRNAs such as polyadenylation, capping, and splicing^3^. High-throughput sequencing and bioinformatics analysis have enabled the identification of a significant number of lncRNAs in various species. Studies of lncRNA functions have been carried out extensively in humans and other model organisms such as zebrafish, mice, yeast, roundworm and fruit flies. Based on studies done in humans and model organisms, scientist discovered that lncRNAs exert their functions by a range of mechanisms. For instance, studies done in human cells showed that lncRNAs regulate gene expression by sequestering miRNAs and transcription factors^4,5^. Mammalian lncRNA *HOTAIR* was reported to perform its function by recruiting chromatin modifying proteins to regulate histone modifications in certain genomic loci^6^. Meanwhile, studies in *Drosophila melanogaster* (*D*. *melanogaster*) provided insights on lncRNA functions in neurogenesis^7^ and spermatogenesis^8^.

*Aedes aegypti* (*Ae*. *aegypti*), a blood-sucking mosquito, is the principal vector responsible for replication and transmission of arboviruses such as dengue (DENV), Chikugunya (CHIKV), and Zika (ZIKV) virus. Functional studies of lncRNAs in non-model organism including *Ae*. *aegypti* are somewhat limited. Previous studies in *Ae*. *aegypti* suggest that lncRNAs are involved in host-virus interaction. For example, RNAi-mediated knockdown of one lncRNA candidate (lincRNA_1317) in *Ae*. *aegypti* resulted in higher viral replication^9^. Additionally, it was reported that lncRNAs were differentially expressed in ZIKV-infected mosquitoes^10^. However, these previous studies are simply descriptive and do not experimentally prove direct interaction or specific mechanisms of lncRNA functions. Although lncRNAs have been systematically identified in many organisms, most lncRNAs have not been functionally characterised.

Recently, the latest version of *Ae*. *aegypti* genome (AaegL5) was released. The assembly was up to chromosome-length scaffolds, which is more contiguous than the previous AaegL3 and AaegL4 assemblies^11^. This prompted us to perform novel lncRNA identification and characterisation using the latest genome release. Previous study^9^ reported that a total of 3,482 intergenic lncRNA (lincRNA) was identified in *Ae*. *aegypti*. However, the identification was performed using previous version of *Ae*. *aegypti* genome (AaegL3), and only lncRNAs located in intergenic regions were annotated.

Here, we report genome-wide identification and characterisation of lncRNAs in *Ae*. *aegypti*. In this study, we defined a high-confident set of 4,689 novel lncRNA transcripts, of which 2,064, 2,076, and 549 were intergenic, intronic, and antisense respectively. We then characterised many features of the newly identified lncRNAs. These features include transcript structures, conservation, and developmental expression. Collectively, genome-wide annotation and characterisation of *Ae*. *aegypti* lncRNAs provide valuable resources for future genetics and molecular studies in this harmful mosquito vector.

## Identification of lncRNA

**Figure 1:**
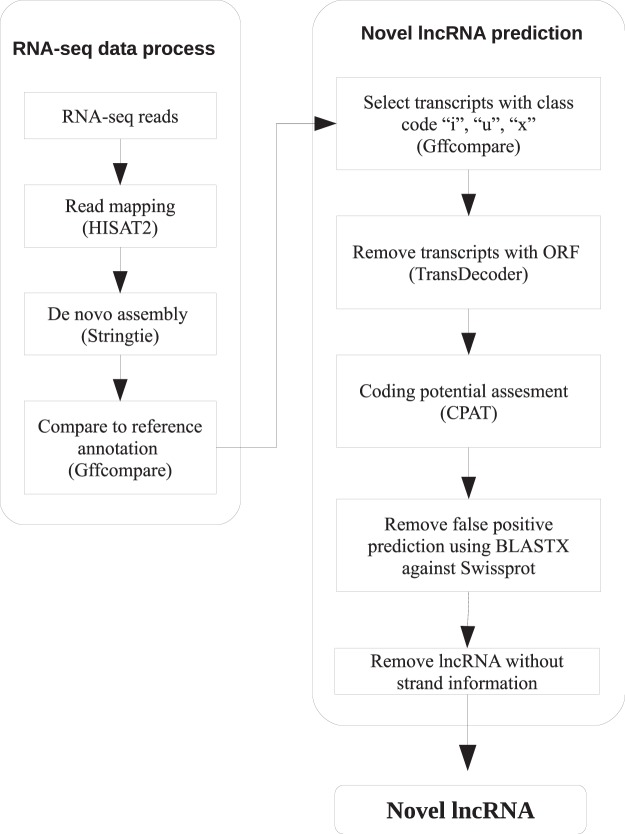
Overview of lncRNA identification pipeline. lncRNA identification pipeline is composed of two processes: RNA-seq data process and novel lncRNA prediction. Briefly, cleaned reads were mapped to the genome using HISAT2 followed by transcriptome assembly by Stringtie. The assembled transcriptome was then compared to reference annotation using Gffcompare. Only transcripts with class code “i”, “u”, and “x” were selected for downstream analysis. A series of protein-coding assessment was performed using TransDecoder, CPAT, and BLASTX. Transcripts having protein-coding potential were removed. Transcripts that did not have strand information were also removed. Transcripts that passed all criteria set in the pipeline were classified as novel lncRNAs.

To perform lncRNA prediction, we used a total of 117 RNA-seq libraries derived from *Ae*. *aegypti* mosquito and Aag2 cell, a widely used *Ae*. *aegypti* derived cell line^12^. An overview of lncRNA identification pipeline can be found in Fig. 1. The pipeline developed in this study was adapted with few modifications from previous reports^9,13,14^. Briefly, each dataset (both paired-end and single-end) was individually mapped using HISAT2^15^. The resulting alignment files were used for transcriptome assembly, and the assemblies were merged into a single unified transcriptome. Both transcriptome assembly and merging were performed using Stringtie^16^. Then, we used Gffcompare to annotate and compare the unified transcriptome assembly with reference annotation (AaegL5.1, VectorBase). We classified lncRNA transcripts based on their position relative to annotated genes derived from AaegL5.1 assembly (VectorBase). We only selected transcripts with class code “i”, “u”, and “x” that denote intronic, intergenic, and antisense to reference genes for downstream analysis.

To get confident lncRNA transcripts, we performed multiple steps of filtering transcripts having coding potential or open-reading frame (Fig. 1). The steps involved TransDecoder^17^, CPAT^18^ and finally BLASTX. We then removed lncRNA candidates that did not have strand information. Detailed description on the prediction analysis and parameter used can be found in Material and Methods section. Using this pipeline, we identified a set of 4,689 novel lncRNA transcript isoforms derived from 3,621 loci. Of these 4,689 lncRNA transcripts, 2,064 and 2,076 were intergenic and intronic respectively, while the remaining 549 transcripts were antisense to reference genes. Currently, AaegL5.1 annotation catalogs 4,155 lncRNA transcripts. Here, we provided another set of 4,689 lncRNA transcripts, making a total of 8,844 lncRNAs in *Ae*. *aegypti*. Genomic coordinates of novel lncRNAs can be found in S1 Data.

## Characterisation of ***Ae***. ***aegypti*** lncRNA

**Figure 2:**
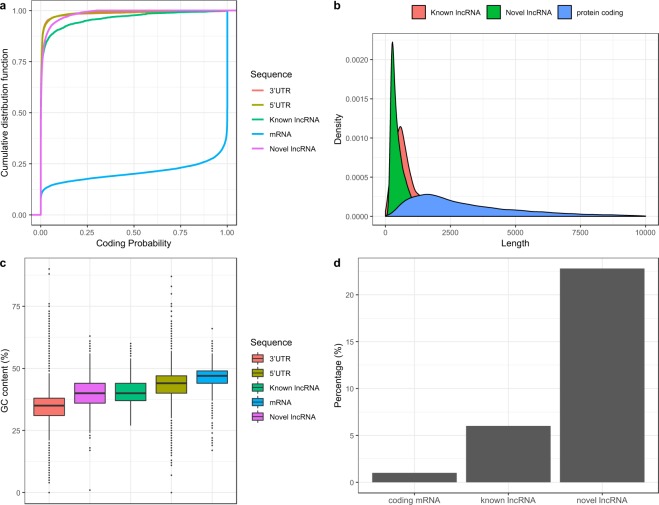
Characterization of *Ae aegypti* lncRNA. (**a**) Coding probability of lncRNA, 3′UTR, 5′UTR and mRNA. (**b**) Sequence length distribution of lncRNA and mRNA. (**c**) GC content. (**d**) Repeat content.

To examine whether lncRNAs identified in this study exhibit typical characteristics observed in other species^13,19–21^, we analysed features such as coding potential, sequence length, GC content and sequence conservation with closely related species. Since lncRNAs are strictly defined by their inability to code for protein, we determined coding probability of our newly identified lncRNAs and compared them with known lncRNA, 3′UTR, 5′UTR, and protein-coding mRNA. We discovered that, similar to other non-coding sequence such as known lncRNA, 3′UTR, and 5′UTR, our novel lncRNA transcripts have extremely low coding probability when compared to protein-coding mRNA (Fig. 2a). Beside that, we found that both novel and known lncRNAs (provided by AaegL5.1 annotation) were shorter than protein-coding transcripts (Fig. 2b). Mean length of novel and known lncRNAs was 825.4 bp and 745.6 bp respectively, while protein-coding mRNA has an average length of 3330 bp.

Similar to previous reports^9,13^, we observed that lncRNAs identified in this study had slightly lower GC content compared to protein-coding mRNAs (Fig. 2c). For instances, mean GC content of novel lncRNA and mRNA was 40.1% and 46.4% respectively. Known lncRNA, on the other hand, had relatively similar mean GC content to novel lncRNA (40.8%), while average GC of 5′UTR and 3′UTR sequence was 43.1% and 34.6% respectively. Overall, GC content of non-coding sequence was relatively lower than coding sequence.

To determine the conservation level of *Ae*. *aegypti* lncRNAs, we performed BLASTN against other insect genomes including *Aedes albopictus* (*Ae*. *albopictus*), *Culex quinquifasciatus* (*C*. *quinquifasciatus*), *Anopheles gambiae* (*An*. *gambiae*), and *D*. *melanogaster*. Bit score derived from BLASTN algorithm was used to determine the level of sequence similarity of Ae. aegypti lncRNAs with previously mentioned insect genomes^9^. Similarly, we also performed BLASTN of *Ae*. *aegypti* protein-coding mRNA for comparison. We discovered that both lncRNAs and protein-coding mRNAs displayed high degree of sequence similarity with Ae. albopictus, suggesting that they were presumably genus specific (Supplementary Fig. 1). In general, compared to protein-coding mRNA, *Ae*. *aegypti* lncRNAs exhibited lower sequence conservation.

It was reported that, in contrast to coding gene, lncRNAs harbour higher composition of repeat elements^22,23^. To test if similar occurrence held true in *Ae*. *aegypti*, we determined the fraction of repeat element in the exons of lncRNAs and protein-coding mRNAs. As expected, we discovered that more than 20% of nt of novel lncRNAs were made up of repeat elements (Fig. 2d). Meanwhile, 6% and 1% of known lncRNAs and protein-coding mRNAs were composed with repeats. Taken together, *Ae*. *aegypti* lncRNAs shared many characteristics with other species: relatively short in length, relatively lower GC content, and higher repeat content.

## Developmental expression of ***Ae***. ***aegypti*** lncRNAs

**Figure 3:**
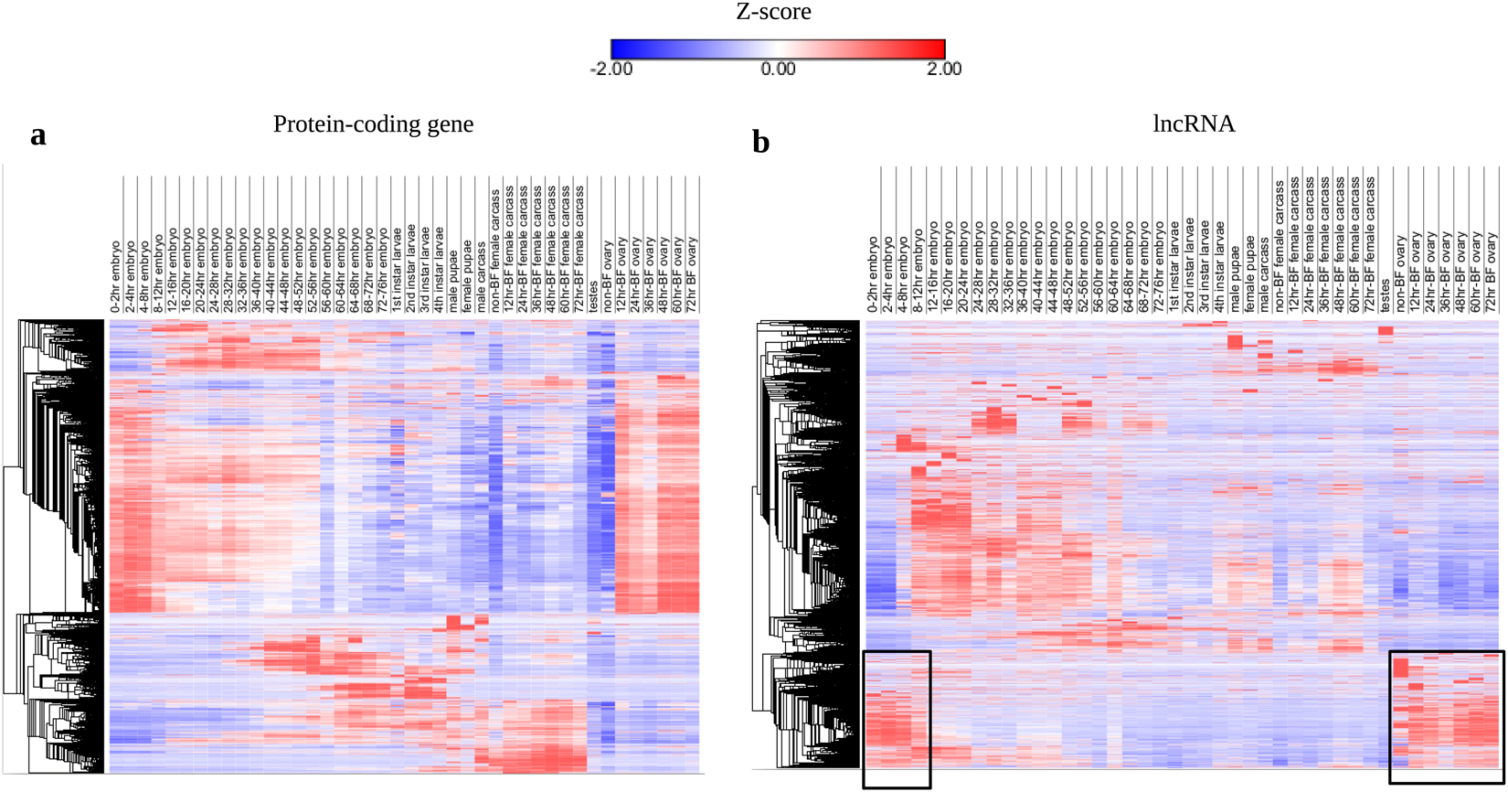
Hierarchical clustering of protein-coding and lncRNA expression. Hierarchical clustering of protein-coding (**a**) and lncRNA (**b**) across developmental stages. Hierarchical clustering analysis was done in Morpheus (https://software.broadinstitute.org/morpheus) based on Pearson correlation of z-scores of lncRNA and protein-coding genes. Boxes in (**b**) indicate a subset of lncRNAs that are presumably maternally inherited.

To examine the developmental expression of *Ae*. *aegypti* lncRNAs, we analysed public dataset (SRP026319) which provided RNA-seq data of specific developmental stages, ranging from early embryo up to adult mosquitoes. These developmental stages include specific time interval of embryonic development, larval stages, sex-biased expression of male versus female pupae and carcass, blood-fed versus nonblood-fed ovary and female carcass, and testes-specific expression. Similar to protein-coding genes, *Ae*. *aegypti* lncRNA genes exhibited stage-specific (embryo, larva, and pupal stages), sex-specific, and blood-fed versus nonblood-fed (ovary and female carcass) expression pattern (Fig. 3). In addition, each time point in the development had distinct lncRNA expression pattern. For instance, there was a subset of lncRNAs that were highly enriched during early embryonic development (0–8 hour embryo) as compared to later time points (8–76 hour embryo). A total of 1,848 lncRNA genes consisting of 24.7% of the total expressed lncRNAs were specifically highly expressed at this early embryonic stage. Interestingly, these early embryo-specific lncRNAs were also highly expressed in blood-fed ovary (Fig. 3). Therefore, our hierarchical clustering statistics suggests that these lncRNAs are maternally inherited, implying that they possess essential roles in early embryonic development.

**Figure 4:**
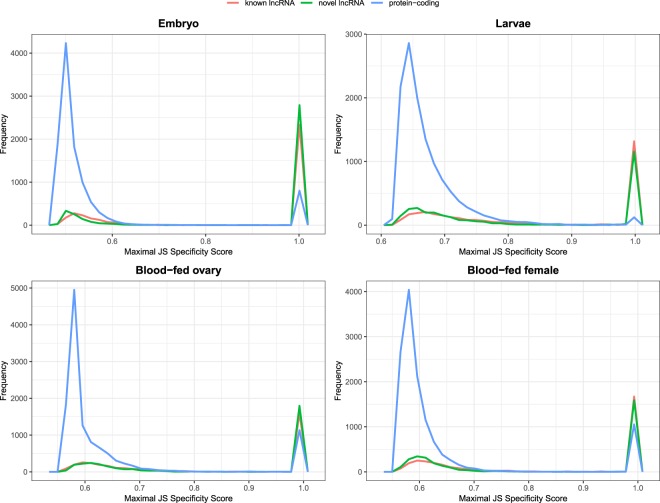
Maximal JS specificity score. Distribution of maximal JS specificity score of lncRNAs and protein-coding genes in embryo, larvae, blood-fed ovary, and blood-fed female mosquitoes. In all stages, the frequency of lncRNA transripts (known and novel) having score of 1 is higher than protein-coding transcripts.

Previous report showed that lncRNAs displayed a more temporally specific expression pattern than protein-coding genes^14^. To test whether or not this is true for *Ae*. *aegypti* lncRNAs, we computed specificity score of each lncRNA using Jensen-Shannon (JS) score^14,24^. The JS score ranged from 0 to 1, with 1 indicated perfect specificity. Here, we computed JS score across two stages namely embryo and larvae, and two conditions which were blood-fed ovary and female carcass, all of which were sampled in a timely fashion. In all four time-course samples namely embryo, larvae, blood-fed female carcass and ovary, we discovered that novel and known lncRNAs had higher JS specificity score in all four samples (Fig. 4). Meanwhile, fraction of protein-coding genes having JS score of 1in all four samples were lower than lncRNAs (Fig. 4). Although lncRNAs had higher developmental and temporal specificity, across all four time-course samples being analysed, the overall expression of lncRNAs was lower than that of protein-coding genes (Supplementary Fig. 2).

**Figure 5:**
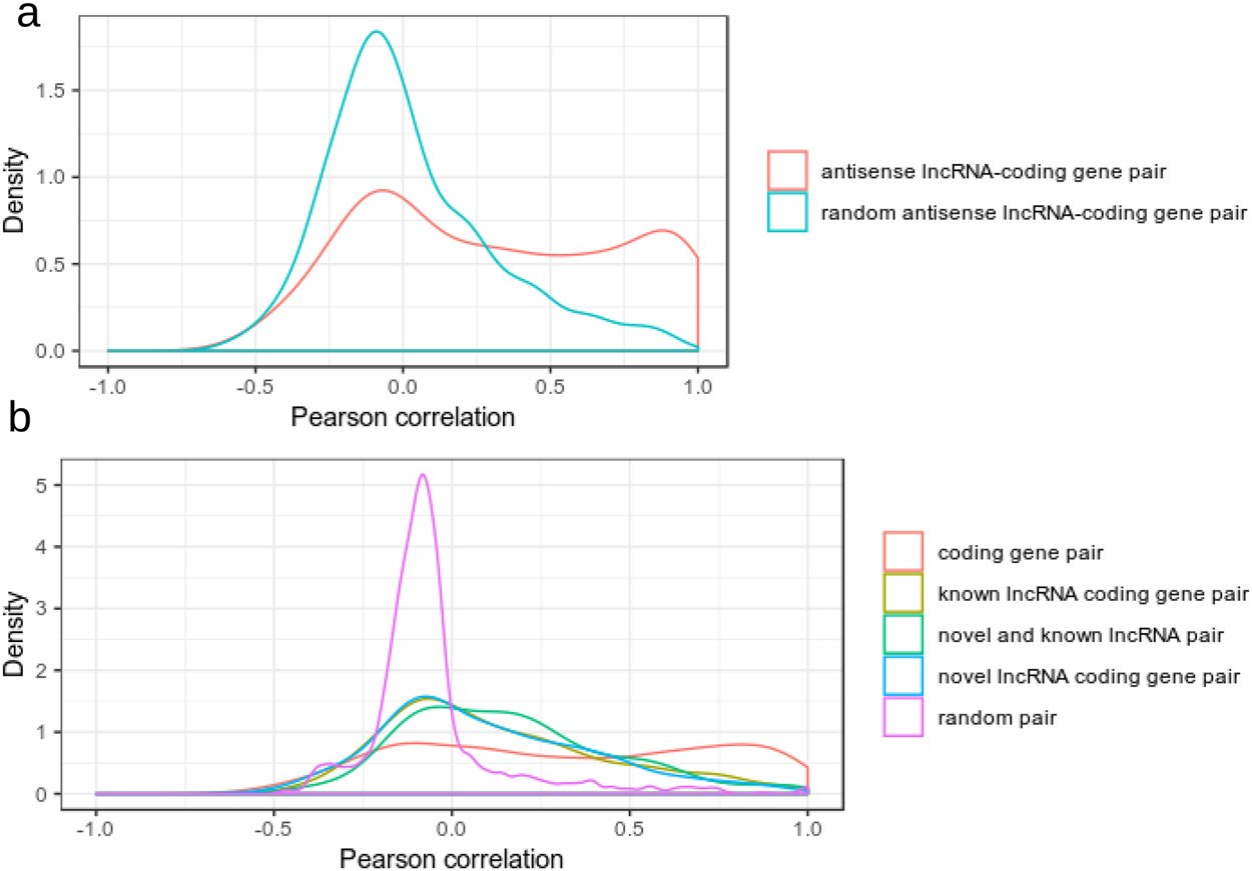
Correlation of expression analysis. (**a**) Pearson correlation of antisense lncRNA expression with its corresponding genes compared to that of antisense lncRNA pair with random protein-coding gene (**b**) Pearson correlation of gene expression within 10 kb from each other. Pearson correlation was computed using R software.

Previous studies have shown that lncRNAs are favourably resided in close proximity to genes with developmental functions^25,26^. In addition, this close physical proximity of lncRNAs and protein-coding genes resulted in their expression to be strongly correlated. We asked whether *Ae*. *aegypti* lncRNAs showed correlation in expression between neighboring protein-coding genes. We also examined the correlation in expression of lncRNAs that were antisense to protein-coding genes. By analysing the expression of 42 developmental samples (Fig. 3), we discovered that fraction of positively correlated (Pearson correlation, p-value < 0.05) antisense lncRNA-coding gene pair was higher than that of randomly assigned antisense lncRNA-coding gene pair (Fig. 5). Analysis of neighboring genes (within 10 kb) revealed that the expression of lncRNAs and their nearest neighboring genes showed a slightly higher degree of correlation compared to random gene pairs. In both cases of random neighbouring pair and random antisense lncRNA-coding gene pair, the majority of the pairs had correlation near to zero value (Fig. 5).

## Discussion

The field of lncRNA has become increasingly important in many areas of biology particularly infectious disease, immunity, and pathogenesis^9,13,20,27^. High-throughput sequencing combined with bioinformatics enable scientists to uncover comprehensive repertoire of lncRNA in many species. Here, we present a comprehensive lncRNA annotation using the latest genome reference of *Ae*. *aegypti* (AaegL5). Due to the recent release of *Ae*. *aegypti* genome (AaegL5) equipped with improved gene set annotation^11^, we decided to perform lncRNA identification using this latest genome reference. Unlike previous annotation that mainly focused on *Ae*. *aegypti* intergenic lncRNA, here, we also annotated lncRNAs that reside within the introns, and lncRNAs that are antisense to reference genes.

Similar to previous reports^9,13,14,27,28^, we discovered that lncRNAs identified in our study exhibited typical characteristics of lncRNAs found in other species including vertebrates^23^. Such characteristics are lower GC content, shorter in length, high repeat content, and low sequence conservation even among closely related species. GC content differs greatly across the genome. Regions of the genome that encode protein usually have higher GC content compared to noncoding regions^29^. In the current study, we confirmed that the GC content of our predicted lncRNAs was lower than coding sequences. One of the common characteristics of lncRNAs across species is shorter in length. Unlike protein-coding mRNAs, lncRNAs do not have ORF, start codon, stop codon, 5′UTR, and 3′UTR. This may be the reason why lncRNAs are generally shorter than protein-coding mRNAs. Another common characteristic of lncRNA is high repeat content. In this study, both known and novel lncRNAs have higher repeat content than protein-coding genes. We observed that the percentage of repeats in novel lncRNAs is much higher than known lncRNAs. This discrepancy is mainly because of different methods used for noncoding RNA annotation. For instance, known lncRNAs were originally from the reference annotation of *Ae*. *aegypti* genome (AaegL5.1), which was derived from NCBI standard annotation pipeline. Meanwhile, our lncRNA prediction pipeline was based on expression data and transcript assembly. Besides, standard genome annotation procedure requires repeat masking before gene finding^30^. lncRNAs in many species including *Ae*. *aegypti* do not exhibit the same conservation pattern as protein-coding genes^9,13,23,24,26^. This makes functional prediction of lncRNAs to be challenging. However, lack of conservation does not necessarily mean lack of function. Studies such as lncRNA-mRNA/protein interaction or loss and gain of functions experiments are crucial to uncover the functional roles of lncRNAs.

Analysis of *Ae*. *aegypti* developmental expression revealed that the expression of lncRNAs is highly temporally specific relative to that of coding genes. In other words, lncRNAs have a much narrower time window in expression than coding genes. Therefore, we hypothesize that lncRNAs in *Ae*. *aegypti* may act as time-specific tuners in regulating the timing of developmental transition. In general, *Ae*. *aegypti* lncRNAs were expressed at lower levels than protein-coding genes. Although lncRNAs are lowly expressed, their high specificity in expression suggests that they potentially perform specific biological functions in a specific stage of development at a specific time point. Future investigation of stage-specific and temporally specific lncRNAs defined in our study may elucidate their functional roles in *Ae*. *aegypti* development. Gene expression study of embryonic neurogenesis in *D*. *melanogaster* revealed a set of conserved lncRNAs that display strict tissue specificity and spatiotemporal expression^7^. Aside from embryogenesis, a set of testis-specific *Drosphila* lncRNAs are required for spermatogenesis. Knock-out of these lncRNAs using CRISPR/Cas9 system resulted in loss of male fertility, and developmental defects in late spermatogenesis^8^. In this study, we provided an evidence of maternal inheritance of *Ae*. *aegypti* lncRNAs. This further corroborated previous findings that highlighted the importance of lncRNAs in insect embryogenesis, metamorphosis, and development^13,14,27,31^. We observed a fraction of lncRNAs that was highly expressed in blood-fed ovary, and the expression persisted up to 8–12 hour embryonic stage. This narrow time window in early embryo was related to maternal-zygotic transition stage^32^. Since transcription from the zygotic genome has not been activated during early embryonic stage, these highly expressed lncRNAs must be maternally provided; suggesting that they might play roles in basic biosynthesis processes in the early embryo, specification of initial cell fate and pattern formation. Meanwhile, *Ae*. *aegypti* lncRNAs expressed at later embryonic, larval and pupal stages are potentially responsible in organogenesis. In summary, we provided a comprehensive genome-wide annotation and characterisation of *Ae*. *aegypti* lncRNAs. We hope that the results from this study will provide valuable resource for future studies on lncRNA functions in *Ae aegypti*.

## Materials and Methods

### RNA-seq data preparation

A total of 117 publicly available RNA-seq datasets were downloaded from NCBI Sequence Reads Archive (SRA) and ArrayExpress with accession numbers SRP173459, SRP041845, SRP047470, SRP046160, SRP115939, E-MTAB-1635, SRP035216, SRP065731, SRP065119, SRA048559, PRJEB13078^10,33–38^. List of the 117 RNA-seq libraries used in this study can be found in S2 Data. Adapters were removed using Trimmomatic version 0.38^39^, and reads with average quality score (Phred Score) above 20 were retained for downstream analysis.

### Mapping of RNA-seq reads against the *Ae*. *aegypti* reference genome

Each library (both paired-end and single-end) was individually mapped against *Ae*. *aegypti* genome (AaegL5) using HISAT2 version 2.1.0^15^. HISAT is considered to be faster with equal or better accuracy than other spliced aligner methods such as Tophat^40^ and STAR^41^. Therefore, the use of HISAT as aligner is feasible, especially because large number of high-depth RNA-seq reads are required for lncRNA prediction. We individually mapped each RNA-seq library to the reference genome because different libraries have different properties such as library type (single-end or paired-end) and strandedness (forward, reverse or unstranded). The parameters used in HISAT2 were adjusted according to the library type.

### Transcriptome assembly

We used Stringtie version 1.3.2^16^ to perform transcriptome assembly. Compared to other transcript assembly softwares such as Cufflinks^42^ and Scripture^43^, StringTie has been shown to produce more comprehensive and accurate transcriptome reconstruction and quantification from RNA-seq data^16^. We used reference annotation file of AaegL5 (VectorBase) to guide the assembly. We set the minimum assembled transcript length to be 200 bp. Then, the output gtf files were merged into a single unified transcriptome using Stringtie merge^16^. Only input transcripts of more than 1 FPKM and TPM were included in the merging. Then, we compared the assembled unified transcript to a reference annotation of AaegL5 (VectorBase) using Gffcompare (https://github.com/gpertea/gffcompare). For the purpose of lncRNA prediction, we only retained transcripts with class code “i”, “u”, and “x”.

### Novel lncRNA prediction

For the purpose of lncRNA prediction, we only retained transcripts with class code “i”, “u”, and “x”. The transcripts were then subjected to coding potential prediction. We used TransDecoder^17^ to identify transcripts having open-reading frame (ORF), and those having ORF were discarded. The remaining transcripts were then subjected to a coding potential assessment toll (CPAT)^18^. CPAT, an alignment-free method, uses logistic regression model generated from sequence features including ORF size, ORF coverage, Fickett TESTCODE statistics, and hexamer usage bias^18^. Besides, CPAT has been optimized for lncRNA prediction in insect model, *D*. *melanogaster*, with high sensitivity (0.96) and specificity (0.97)^18^. We set the same cut-off as previous study in *Ae*. *aegypti* which is less than 0.3^9^. Transcripts having coding potential more than 0.3 were discarded. To exclude false positive prediction, we used BLASTX against Swissprot database, and transcripts having E-value of less than 10^–5^ were removed. We also removed novel transcripts that do not have strand information. Without strand information it is difficult to correctly determine where the RNA transcripts originate from. Moreover, since the expression of lncRNAs tend to be correlated with neighbouring genes, it is imperative to have information on their strand in the genome.

### Transcript quantification and expression

We used Salmon version 0.10.1^44^ to quantify the expression of transcripts. We used TPM value computed by Salmon for downstream analysis. Salmon was used for transcript abundance quantification in this study due to its rapidness and accuracy since the algorithm is able to correct for fragment GC content bias^44^.

### Coding potential and GC content analyses

5′ UTR, 3′UTR, and known lncRNA sequence were downloaded from VectorBase using BioMart tool. Coding potential assessment was done using CPAT^18^. Meanwhile, GC content of each sequence was evaluated using EMBOSS geecee program^45^.

### Sequence conservation analysis of *Ae*. *aegypti* lncRNAs

We used previously described method^9^ to evaluate sequence conservation of *Ae*. *aegypti* lncRNAs. The genomes of *Ae*. *albopictus* (Assembly: AaloF1), *C*. *quinquifasciatus* (Assembly: CpipJ2), and *An*. *gambiae* (Assembly: AgamP4) were downloaded from VectorBase. The genome of *D*. *melanogaster* (Assembly: BDGP6.22) was downloaded from ENSEMBL database^46^. Sequence similarity of *Ae*. *aegypti* lncRNAs were searched against these insect genomes with BLASTN (E-value < 10^−5^). Bitscore was used to evaluate the level of sequence similarity with previously mentioned insect genomes.

### Expression specificity analysis

JS tissue-specificity score of each lncRNA was computed as previously described^24^. In the current study, we calculated specificity score for each gene using MATLAB version R2018b using the formula given from previous work^24^.

## Supplementary information

Supplementary figure

S2 Data

S1 Data
