# Supplementary material for: Systematic identification and characterization of Aedes aegypti long noncoding RNAs (lncRNAs)
Source: Sci Rep. 2019 Aug 21;9:12147. doi: 10.1038/s41598-019-47506-9 (PMC6704130; doi:10.1038/s41598-019-47506-9)
Supplement: Supplementary file 1 — Supplementary figure [file 41598_2019_47506_MOESM1_ESM.pdf]

Supplementary information

Systematic identification and characterization of *Aedes aegypti* long noncoding RNAs (lncRNAs)

Azali Azlan[1], Sattam M. Obeidat[1], Muhammad Amir Yunus[2] and Ghows Azzam[1]*

[1]*School of Biological Sciences, Universiti Sains Malaysia, 11800 Penang, Malaysia*

[2]*Infectomics Cluster, Advanced Medical & Dental Institute, Universiti Sains Malaysia, Bertam, 13200 Kepala Batas, Pulau Pinang, Malaysia.*

*Corresponding author

Email: ghows@usm.my (G.A)

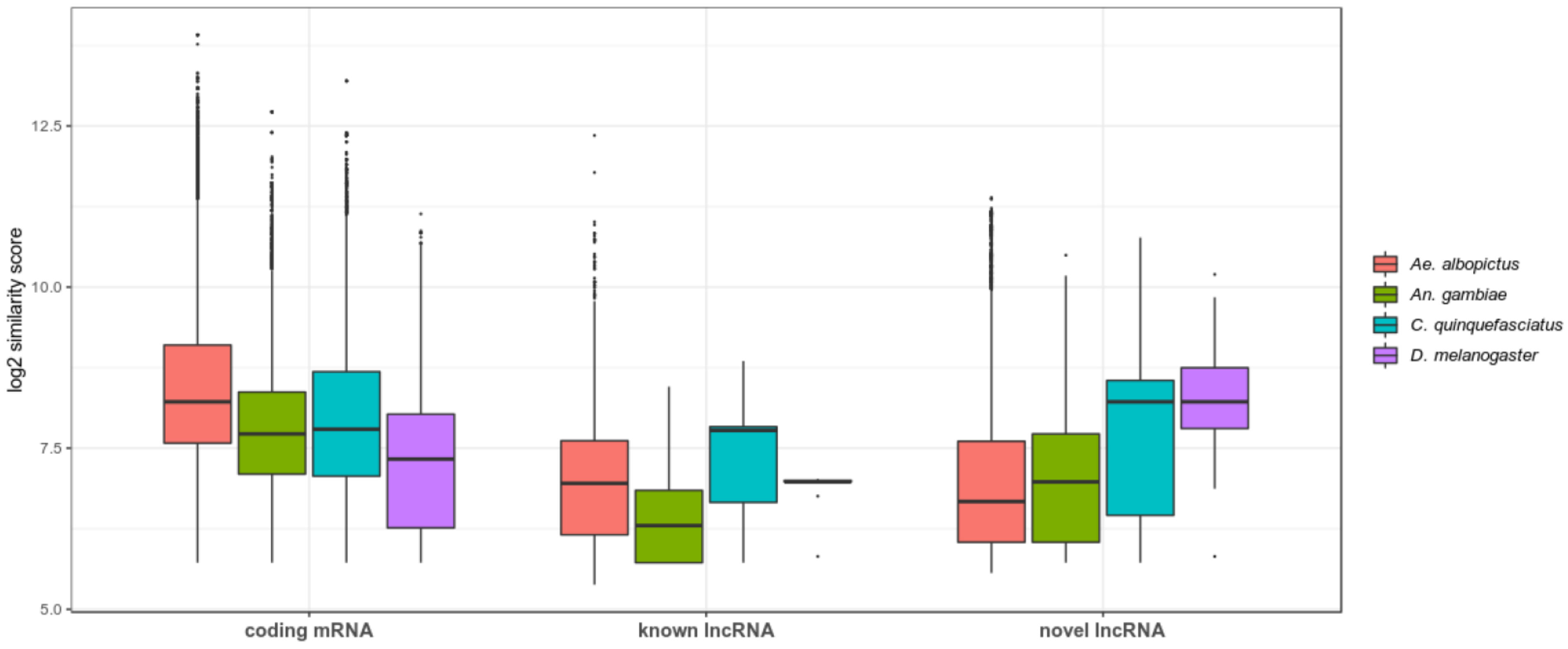

S1 Fig. Similarity bit score of lncRNA and mRNA with closely related insect genomes. *Ae. aegypti* coding mRNAs, known and novel lncRNAs were aligned to insect genomes using BLASTN algorithm. Bit score of the alignment was used as indicator to determine the level of sequence similarity.

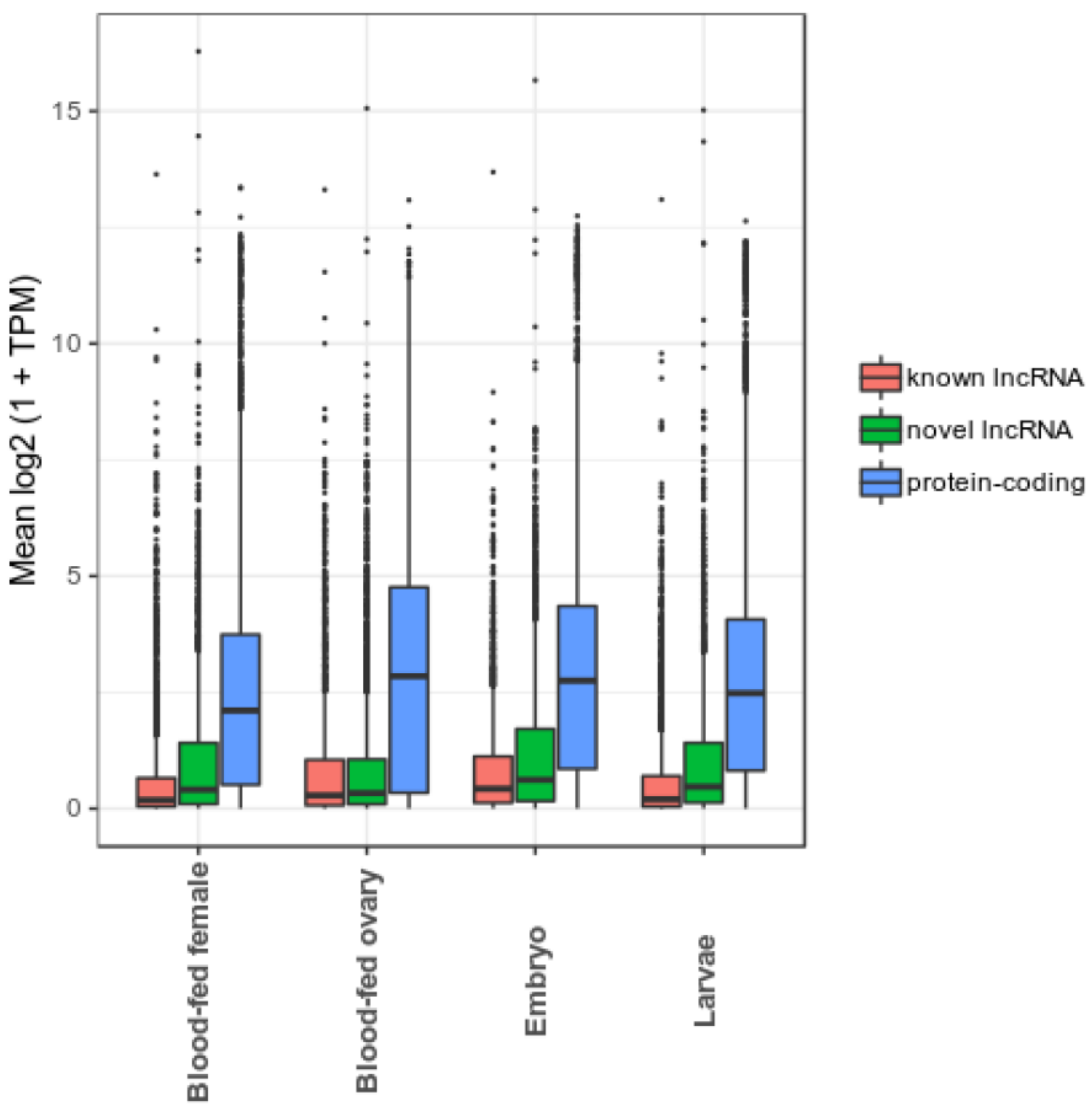

S2 Fig. Distribution of lncRNA and coding gene expression in embryo, larvae, blood-fed ovary and female carcass. Each stage (embryo, larvae, blood-fed ovary and blood-fed female) was sampled in a timely manner. Here, we took the mean expression of protein-coding and lncRNAs genes in each stage. Across all stages, the overall expression of lncRNAs was lower than protein-coding genes.
