# Supplementary material for: Systematic identification and characterization of Aedes aegypti long noncoding RNAs (lncRNAs)
Source: Sci Rep. 2019 Aug 21;9:12147. doi: 10.1038/s41598-019-47506-9 (PMC6704130; doi:10.1038/s41598-019-47506-9)
Supplement: Supplementary file 2 — S2 Data [file 41598_2019_47506_MOESM2_ESM.docx]

Supplementary Data S2

Systematic identification and characterization of *Aedes aegypti* long noncoding RNAs (lncRNAs)

**Azali Azlan^1^, Sattam M. Obeidat^1^, Muhammad Amir Yunus^2^ and Ghows Azzam^1^***

^1^*School of Biological Sciences, Universiti Sains Malaysia, 11800 Penang, Malaysia*

^2^*Infectomics Cluster, Advanced Medical & Dental Institute, Universiti Sains Malaysia, Bertam, 13200 Kepala Batas, Pulau Pinang, Malaysia.*

*Corresponding author

Email: ghows@usm.my (G.A)

SRR8318512

SRR8318513

SRR8318515

SRR8318516

SRR8318517

SRR3680431

SRR1283006

SRR3680433

SRR3680434

SRR1585314

SRR1585315

SRR1585316

SRR1585317

SRR1585318

SRR1585319

SRR1568262

SRR1568263

SRR1568264

SRR1578251

SRR1578254

SRR1578255

SRR1578250

SRR1578252

SRR1578256

SRR5955010

SRR5955011

SRR5955012

SRR5955014

SRR5955015

SRR5955016

SRR5955017

SRR5955018

SRR5955019

SRR5955020

SRR5955021

SRR5955022

SRR5955023

SRR5955024

SRR5955025

SRR5955026

SRR5955027

ERR266245

ERR266246

ERR266249

ERR266248

ERR266250

ERR266256

ERR266251

ERR266253

ERR266254

ERR266255

ERR266252

SRR1103950

SRR1103952

SRR1103953

SRR1103954

SRR1103955

SRR1103956

SRR1103957

SRR1103958

SRR1103959

SRR1103960

SRR1103961

SRR1103962

SRR1103963

SRR1103964

SRR1103965

SRR1103967

SRR1103968

SRR1103969

SRR1103970

SRR1103971

SRR1103972

SRR1103973

SRR1103975

SRR1103976

SRR1103977

SRR1103978

SRR1103979

SRR1103980

SRR1103981

SRR1103982

SRR2891730

SRR2891731

SRR2891733

SRR2891734

SRR2891735

SRR2891736

SRR2891737

SRR2891738

SRR2891739

SRR2891740

SRR2891741

SRR2891742

SRR2891743

SRR2891744

SRR2891745

SRR2891746

SRR2891747

SRR2891748

SRR2891749

SRR2891750

SRR2891751

SRR2891752

SRR2891753

SRR2891754

SRR2891755

SRR2762246

SRR2761364

SRR388677

SRR388678

SRR388679

SRR388680

SRR388681

SRR388682

SRR388683

ERR1331745
